# NONLINEAR ASSOCIATIONS OF DAILY STRESS REACTIVITY WITH HEALTH AND WELL-BEING

**DOI:** 10.1093/geroni/igac059.1029

**Published:** 2022-12-20

**Authors:** Jonathan Rush, Anthony Ong, Jennifer Piazza, Susan Charles, David Almeida

**Affiliations:** University of Victoria, Victoria, British Columbia, Canada; Cornell University, Ithaca, New York, United States; California State University, Fullerton, Fullerton, California, United States; University of California, Irvine, Irvine, California, United States; The Pennsylvania State University, University Park, Pennsylvania, United States

## Abstract

Research has repeatedly demonstrated that greater affective reactivity to daily stressors is associated with detrimental health outcomes (e.g. inflammation, mortality). However, most research has only considered linear effects, which precludes an examination of whether moderate levels of stress reactivity may be beneficial. Using daily diary data from the National Study of Daily Experiences (N=2,018) we fit multilevel SEMs to simultaneously model daily within-person associations between stress and negative affect (i.e., stress reactivity), and individual differences in the linear and quadratic associations between stress reactivity and life satisfaction, psychological distress, and chronic conditions. Significant quadratic effects were found for each of the three outcomes (estimates=-20.23; 11.49; 20.81, ps<.001, respectively), indicating a U-shaped pattern where both low and high levels of stress reactivity were associated with poorer health, whereas moderate levels of daily stress reactivity predicted better health outcomes. The results suggest that some affective response to daily stressors can be beneficial.

